# The neurocognitive function change criteria after whole-brain radiation therapy for brain metastasis, in reference to health-related quality of life changes: a prospective observation study

**DOI:** 10.1186/s12885-020-6559-3

**Published:** 2020-01-29

**Authors:** Toshimichi Nakano, Hidefumi Aoyama, Hirotake Saito, Satoshi Tanabe, Kensuke Tanaka, Katsuya Maruyama, Tomoya Oshikane, Atsushi Ohta, Eisuke Abe, Motoki Kaidu

**Affiliations:** 1 Department of Radiology and Radiation Oncology, Niigata University Graduate School of Medical and Dental Sciences, 1-757 Asahimachi-dori, Chuo-ku, Niigata, 951-8510 Japan; 2 Department of Radiation Oncology, Niigata University Medical and Dental hospital, Niigata, Japan

**Keywords:** Brain metastasis, Health-related quality of life, Neurocognitive function, Whole brain radiation therapy

## Abstract

**Background:**

We sought to construct the optimal neurocognitive function (NCF) change criteria sensitive to health-related quality of life (HR-QOL) in patients who have undergone whole-brain radiation therapy (WBRT) for brain metastasis.

**Methods:**

We categorized the patients by the changes of NCF into groups of improvement versus deterioration if at least one domain showed changes that exceeded the cut-off while other domains remained stable. The remaining patients were categorized as stable, and the patients who showed both significant improvement and deterioration were categorized as ‘both.’ We examined the clinical meaning of NCF changes using the cut-off values 1.0, 1.5, and 2.0 SD based on the percentage of patients whose HR-QOL changes were ≥ 10 points.

**Results:**

Baseline, 4-month and 8-month data were available in 78, 41 (compliance; 85%), and 29 (81%) patients, respectively. At 4 months, improvement/stable/deterioration/both was seen in 15%/12%/41%/32% of the patients when 1.0 SD was used; 19%/22%/37%/22% with 1.5 SD, and 17%/37%/37%/9% with 2.0 SD. The HR-QOL scores on the QLQ-C30 functional scale were significantly worse in the deterioration group versus the others with 1.0 SD (*p* = 0.013) and 1.5 SD (*p* = 0.015). With 1.5 SD, the HR-QOL scores on the QLQ-BN20 was significantly better in the improvement group versus the others (*p* = 0.033). However, when ‘both’ was included in ‘improvement’ or ‘deterioration,’ no significant difference in HR-QOL was detected.

**Conclusions:**

The NCF cut-off of 1.5 SD and the exclusion of ‘both’ patients from the ‘deterioration’ and ‘improvement’ groups best reflects HR-QOL changes.

## Background

Brain metastases are a common sequela of cancer, occurring in approx. 24–45% of all cancer patients [1]. Neurocognitive function (NCF) is considered to reflect the status of the brain tumor burden as well as the degree of the adverse radiation effect on the brain [2, 3]. The combination of the Hopkins Verbal Learning Test-Revised (HVLT-R), the Trail-Making Test (TMT), and the Control Oral Word Association Test (COWAT) is a standardized neurocognitive battery, proposed by the Response Assessment in Neuro-Oncology (RANO) Group [4].

However, NCF change score criteria are not uniform across studies [5], and the proportion of patients reported to be suffering from neurocognitive deterioration after whole-brain radiation therapy (WBRT) has thus varied widely among studies, from 52 to 91.7% [6, 7]. It is also difficult to interpret whether the deterioration at 3–4 months after radiation is clinically meaningful, because of numerous confounding factors. We examined the validity of SD (1.0, 1.5, or 2.0) [6, 8], which are employed in the previous major trials.

We designed an observational study to address these issues, using successively collected NCF and health-related quality of life (HR-QOL) data in patients who underwent WBRT for brain metastases. Our study objectives were to clarify the appropriate NCF change criteria reflecting clinically meaningful changes in HR-QOL.

This is the first-ever study using the Japanese version of the RANO-proposed battery for the assessment of NCF and HR-QOL.

## Methods

### Study population and treatment

Patients were eligible if they were ≥ 18 years old and had one or more brain metastases and were scheduled to undergo WBRT. The recruitment of patients took place between April 2012 and March 2017. The exclusion criteria were: Karnofsky Performance Status (KPS) < 60, and severe neurological deficits that could interfere with the administration of the NCF and HR-QOL examinations. Written informed consent was obtained from all patients. This study was approved by the Institutional Review Board (Study #1449).

### The assessment of neurocognitive function and health-related quality of life

The patients’ NCF was assessed using the Japanese versions of the HVLT-R, TMT, and COWAT. The HVLT-R consists of three domains, i.e., Total Recall (TR), Delayed Recall (DR), and Delayed Recognition (DRec), which are related to immediate and learning memory, delayed memory, and recognition, respectively. Parts A and B of the TMT (TMT-A) and (TMT-B) assess an individual’s processing speed and executive function, respectively. The COWAT assesses semantic fluency.

We converted the patients’ raw individual test scores into standardized z-scores by using the means and standard deviations (SDs) of individually age- and gender-matched healthy controls [9, 10]. We selected changes in the z-score of ≥1.0, 1.5, and 2.0 of SD to examine how the differences in the cut-off value may affect the HR-QOL values [6, 8, 11].

The patients’ HR-QOL was assessed with two meansures: the European Organization for Research and Treatment of Cancer Quality of Life Questionnaire Core 30 (EORTC QLQ-C30) ver. 3.0 [12] and the Brain Cancer Module (QLQ-BN20) [13]. The QLQ-C30 is composed of one scale measuring an individual’s global health status (GHS), five functional scales (physical, role, social, emotional, and cognitive functioning), and nine symptom scales (fatigue, nausea/vomiting, pain, dyspnea, insomnia, appetite loss, constipation, diarrhea, and financial difficulties). The QLQ-BN20 is a disease-specific module for brain cancer patients, and it consists of 11 symptom scales: future uncertainty, visual disorder, motor dysfunction, communication deficit, headaches, seizures, drowsiness, itchy skin, hair loss, weakness of legs, and bladder control. All raw scores were linearly transformed and scored from 0 to 100 according to the guidelines. A higher score on the GHS scale or the functioning scales indicates higher quality of life, whereas a higher score on the symptom scales indicates poorer quality of life.

We used the patients’ NCF test scores and HR-QOL questionnaire responses obtained at baseline, 4 months, and 8 months after WBRT in this study.

### Statistical analyses

#### At baseline

The patients’ NCF and HR-QOL scores are presented as mean scores. We compared the scores between groups based on age (< 65 years old vs. ≥65 years old), KPS (100–80 vs. 70–60), the patients’ number of brain metastases (1–4 vs. ≥5 or meningeal carcinomatosis, the Graded Prognostic Assessment (GPA) (4.0–2.0 vs. 1.5–1.0) [14], the number of examinations (1: baseline-only data were available vs. ≥2: the baseline plus 4-month data, or the baseline, 4-month, and 8-month data were available), the use of surgical resection for brain metastases, and the systemic therapies prior to WBRT. We used the Mann-Whitney test for the individual NCF domains and the HR-QOL scales.

#### At 4 months and 8 months

##### NCF and HR-QOL

Regarding the domain level, we assigned the changes in the score on each NCF domain over the time periods of the baseline to 4 months and the baseline to 8 months to one of three categories: ‘improvement,’ ‘stable,’ or ‘deterioration.’ Improvement and deterioration were defined as an increase or decrease in the score over the cut-off value (1.0, 1.5, or 2.0 SD). Other changes were defined as ‘stable.’

Regarding the patient level, we classified the patients into four categories: ‘improvement,’ ‘deterioration,’ ‘stable,’ and ‘both.’ ‘Improvement’ and ‘deterioration’ were defined as exhibiting significant improvement or deterioration in NCF on at least one domain while other domains remained stable. The category of ‘stable’ was defined as no significant changes in any domain, whereas the ‘both’ category included the patients who showed both significant improvement and deterioration, in different domains.

Regarding the patients’ HR-QOL, the ≥10-point changes of each scale at 4 months and 8 months were assigned to one of three categories: ‘improvement,’ ‘stable,’ or ‘deterioration’ for individual HR-QOL scales. The same categorization was done for the mean QLQ-C30 functional/symptoms scales and the QLQ-BN20 scales.

##### The relationship of the changes in NCF and HR-QOL at 4 months

We compared the percentages of deterioration and improvement on HR-QOL scales at 4 months post-WBRT between groups at the patient level (deterioration vs. others, deterioration + both vs. others, improvement vs. others, and improvement + both vs. others) using the three cut-off values of 1.0, 1.5, and 2.0 SD. We used Fisher’s exact test to examine the differences in the independent HR-QOL scales and the differences in the means QLQ-C30 functional/symptom scales and QLQ-BN20 scales.

##### The factors related to the deteriorations in NCF and HR-QOL at 4 months

To examine the deterioration of NCF and that of HR-QOL from baseline to 4 months, we compared the percentage of deterioration on the NCF domains, GHS scale, and the means QLQ-C30 functional/symptom scales and QLQ-BN20 scales between groups based on age (< 65 vs. ≥65 years old), GPA (4.0–2.0 vs. 1.5–0.0), the number of examinations (two examinations [baseline and 4 months] vs. three examinations [baseline, 4 months, and 8 months]), intracranial control at 4 months (progressive disease [PD] vs. non-PD), the systemic therapies prior to WBRT, the cytotoxic chemotherapy after WBRT, and the molecular targeted therapy after WBRT, using Fisher’s exact test.

Overall survival was assessed by the Kaplan Meier-method. A *p*-value < 0.05 was considered significant. All statistical analyses were performed with SPSS ver. 25 software.

## Results

### The patients’ characteristics

The characteristics of the 78 consecutive patients at baseline and the 41 patients who underwent the examinations at 4 months are listed in Tables 1 and 2. The examinations compliance at 4 months and 8 months were high at 85% (41/48) and 81% (29/36), respectively. The median survival time (MST) of all patients was 7.3 months (95% confidence interval: 4.2–10.4), 2.6 months (1.8–3.4) in the baseline-only group, 8.2 (6.1–10.3) in the baseline plus 4-month examinations group, and 26.6 months (21.8–31.4) in the baseline, 4-month and 8-month examination group (Additional file 1: Figure S1).

**Table 1:** The characteristics of the 78 patients who underwent whole-brain radiation therapy

Age, yrs.; mean (SD):	
Total	61.8 (11.5)
< 65	45 (58)
≥ 65	33 (42)
Gender:	
Male	43 (55)
Female	35 (45)
Primary cancer:	
Lung, non-small-cell	36 (46)
Breast	12 (15)
Lung, small-cell	6 (8)
Others	24 (31)
KPS:	
100–90	26 (33)
80	24 (31)
70	16 (21)
60	12 (15)
No. of brain metastases:	
Solitary	17 (22)
Oligo (2–4)	16 (21)
Multiple (5 = <)	34 (43)
Meningeal carcinomatosis	11 (14)
GPA:	
4.0–3.0	5 (6)
2.5–2.0	20 (26)
1.5–1.0	32 (51)
0.5–0.0	21 (27)
WBRT schedule:	
35 Gy/14 fr	63 (81)
30 Gy/10 fr	7 (9)
Others	5 (6)
Uncompleted	3 (4)
Treatment:	
WBRT only	47 (60)
WBRT + SRS	11 (14)
WBRT + Surgery (+/−SRS)	20 (26)
Systemic therapies prior to WBRT	
No	23 (29)
Yes	55 (71)
Cytotoxic chemotherapy	50 (64)
Molecular targeted therapy	25 (32)
Hormonal therapy	7 (9)
Immunotherapy	2 (3)
No. of examinations:	
1: BL only	37 (48)
2: BL and 4 mos.	12 (15)
3: BL, 4 mos. and 8 mos.	29 (37)
Examination compliance:%, (/):	
At 4 mos.	85% (41/48)
At 8 mos.	81% (29/36)

Data are *n* (%) unless otherwise noted. *KPS* Karnofsky Performance Status, *GPA* Graded Prognosis Assessment, *WBRT* whole-brain radiotherapy, *SRS* stereotactic radiosurgery, *BL* baseline, *PD* progressive disease

**Table 2:** The characteristics of the 41 patients who underwent the examinations at 4 months

Age, yrs.; mean (SD):	
Total	63.0 (11.4)
< 65	21 (51)
≥ 65	20 (49)
Gender:	
Male	19 (46)
Female	22 (54)
Primary cancer:	
Lung, non-small-cell	24 (59)
Breast	6 (15)
Lung, small-cell	3 (7)
Others	8 (19)
KPS:	
100–90	15 (37)
80	14 (34)
70	7 (17)
60	5 (12)
No. of brain metastases:	
Solitary	10 (24)
Oligo (2–4)	12 (29)
Multiple (5 = <)	14 (34)
Meningeal carcinomatosis	5 (12)
GPA:	
4.0–3.0	4 (10)
2.5–2.0	13 (32)
1.5–1.0	18 (44)
0.5–0.0	6 (14)
WBRT schedule:	
35 Gy/14 fr	36 (88)
30 Gy/10 fr	1 (2)
Others	4 (10)
Treatment:	
WBRT only	20 (49)
WBRT + SRS	7 (17)
WBRT + Surgery (+/−SRS)	14 (34)
Systemic therapies prior WBRT	
No	16 (39)
Yes	25 (61)
Cytotoxic chemotherapy	22 (54)
Molecular targeted therapy	11 (27)
Hormonal therapy	4 (10)
Immunotherapy	1 (2)
Systemic therapies after WBRT	
No	6 (15)
Yes	35 (85)
Cytotoxic chemotherapy	19 (46)
Molecular targeted therapy	19 (46)
Hormonal therapy	2 (5)
Immunotherapy	3 (7)
Intracranial control at 4 mos.	
PD	13 (32)
Non-PD	26 (63)
Unknown	2 (5)

Data are *n* (%) unless otherwise noted. *KPS* Karnofsky Performance Status, *GPA* Graded Prognosis Assessment, *WBRT* whole-brain radiotherapy, *SRS* stereotactic radiosurgery, *BL* baseline, *PD* progressive disease

### The baseline NCF and HR-QOL data

The mean z-score was − 1.46 for the TR, − 1.75 for the DR, − 1.07 for the DRec, − 0.46 for the COWAT, − 1.46 for the TMT-A, and − 1.12 for the TMT-B. The mean score for each HR-QOL scale are provided in Additional file 2: Table S1. Among the factors examined, only poor KPS of 60–70 (vs. 80–100) was associated with significantly poor NCF in two domains. Regarding the HR-QOL (total 26 scales), significantly poor scores were observed in the patients with a KPS of 60–70 on 16 scales, or a GPA of 0–1.5 on 11 scales; age ≥ 65 years on five scales, the availability of examination (baseline-only) in three scales, undergoing surgery in three scales, the systemic therapies prior to WBRT in three scales; and the number of metastases ≥5 or meningeal carcinomatosis on two scales. (Additional file 2: Table S1).

### The NCF and HR-QOL data at 4 months and 8 months

#### The changes in the patients’ NCF and HR-QOL

**Fig. 1:**
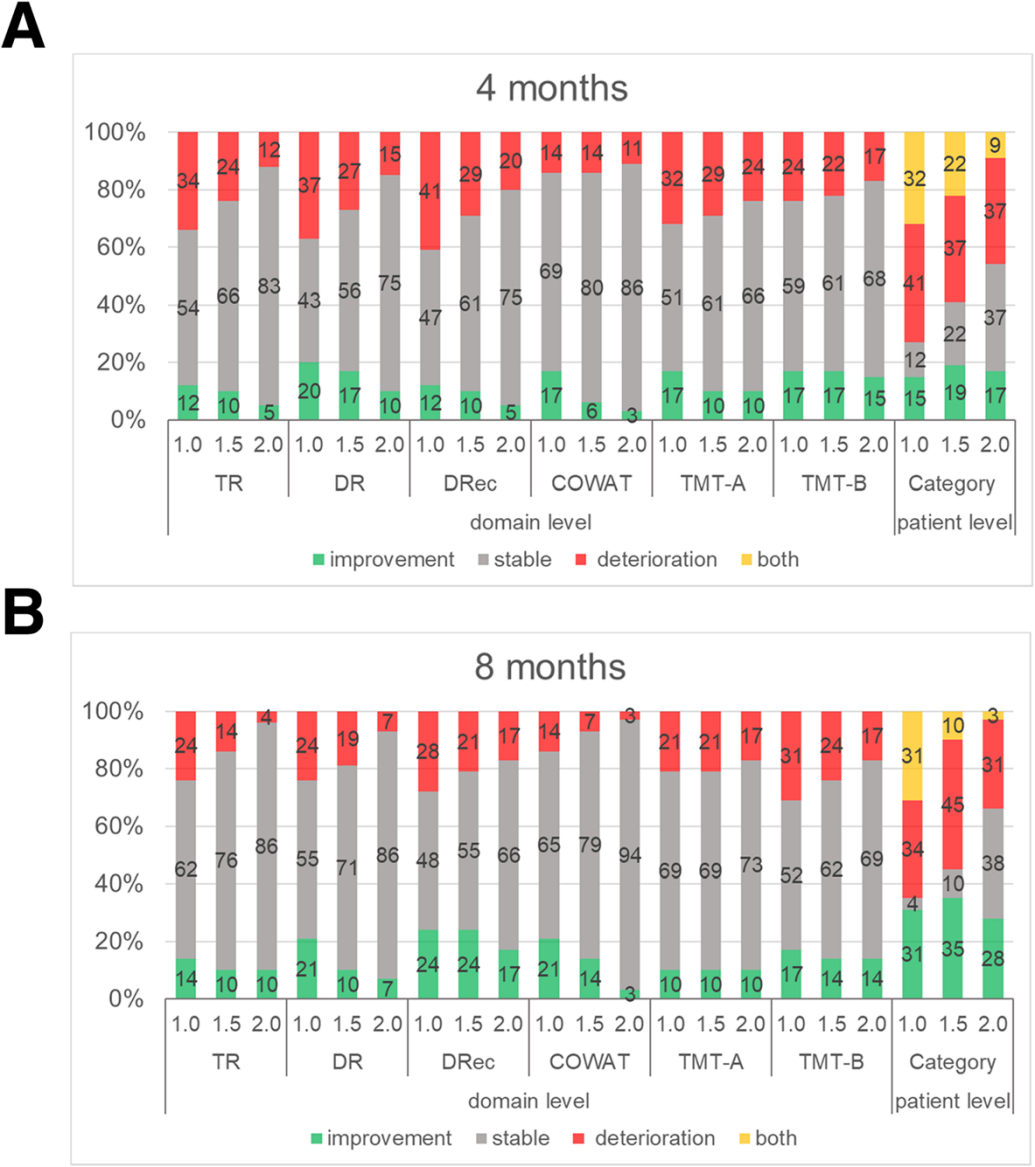
Changes in the patients’ NCF scores from baseline to 4 months (**a**) and from baseline to 8 months (**b**) at the domain level and at the patient level with the use of three cut-off values. TR: Total Recall domain of the Hopkins Verbal Learning Test-Revised (HVLT-R), DR: Delayed Recall domain of the HVLT-R, DRec: the Delayed Recognition domain of the HVLT-R, COWAT: the Control Oral Word Association Test, TMT-A: part A of the Trail-Making Test (TMT), TMT-B: part B of the TMT

At the domain level, regarding the changes in the NCF of the patients at 4 months after they underwent WBRT, when we used the cut-off value of 1.0 SD, NCF improvement was documented in 12–20% (TR/12%, DR/20%, DRec/12%, COWAT/17%, TMT-A/17%, TMT-B/17%) of the patients, stable NCF was observed in 43–69% (TR/54%, DR/43%, DRec/47%, COWAT/69%, TMT-A/51%, TMT-B/59%), and deterioration was exhibited by 14–41% (TR/34%, DR/37%, DRec/41%, COWAT/14%, TMT-A/32%, TMT-B/24%) compared to the baseline. The data at 8 months were 10–24% improvement (TR/14%, DR/21%, DRec/24%, COWAT/21%, TMT-A/10%, TMT-B/17%), 52–69% stable (TR/62%, DR/55%, DRec/48%, COWAT/65%, TMT-A/69%, TMT-B/52%), and 14–31% deterioration (TR/24%, DR/24%, DRec/28%, COWAT/14%, TMT-A/21%, TMT-B/31%). With the cut-off value 1.5 SD, NCF improvement was documented in 6–17% of the patients, stable NCF was observed in 56–80%, and deterioration was observed in 14–29% at 4 months, and the corresponding values at 8 months were 10–24%, 55–79%, and 7–24%, respectively. With the cut-off values of 2.0 SD, these values were 3–15%, 66–86%, 11–24% at 4 months, and 3–17%, 66–94%, and 3–17% at 8 months, respectively (Fig. 1).

At the patient level, the percentage of patients who exhibited deterioration, improvement, both, or were stable with the use of different cut-off values (1.0 SD, 1.5 SD, and 2.0 SD) are summarized in Fig. 1. At 4 months, improvement/stable/deterioration/both was seen in 15%/12%/41%/32% of the patients when 1.0 SD was used as the cut-off, 19%/22%/37%/22% of the patients when 1.5 SD was used, and 17%/37%/37%/9% of the patients when 2.0 SD was used. It is of note that the ‘both’ percentage was reduced from 32% with the cut-off value of 1.0 SD to 22% with 1.5 SD, and further reduced to 9% with 2.0 SD. At 8 months, improvement/stable/deterioration/both was seen in 31%/4%/34%/31% of the patients when 1.0 SD was used as the cut-off, 35%/10%/45%/10% with 1.5 SD, and 28%/38%/31%/3% with 2.0 SD.

**Fig. 2:**
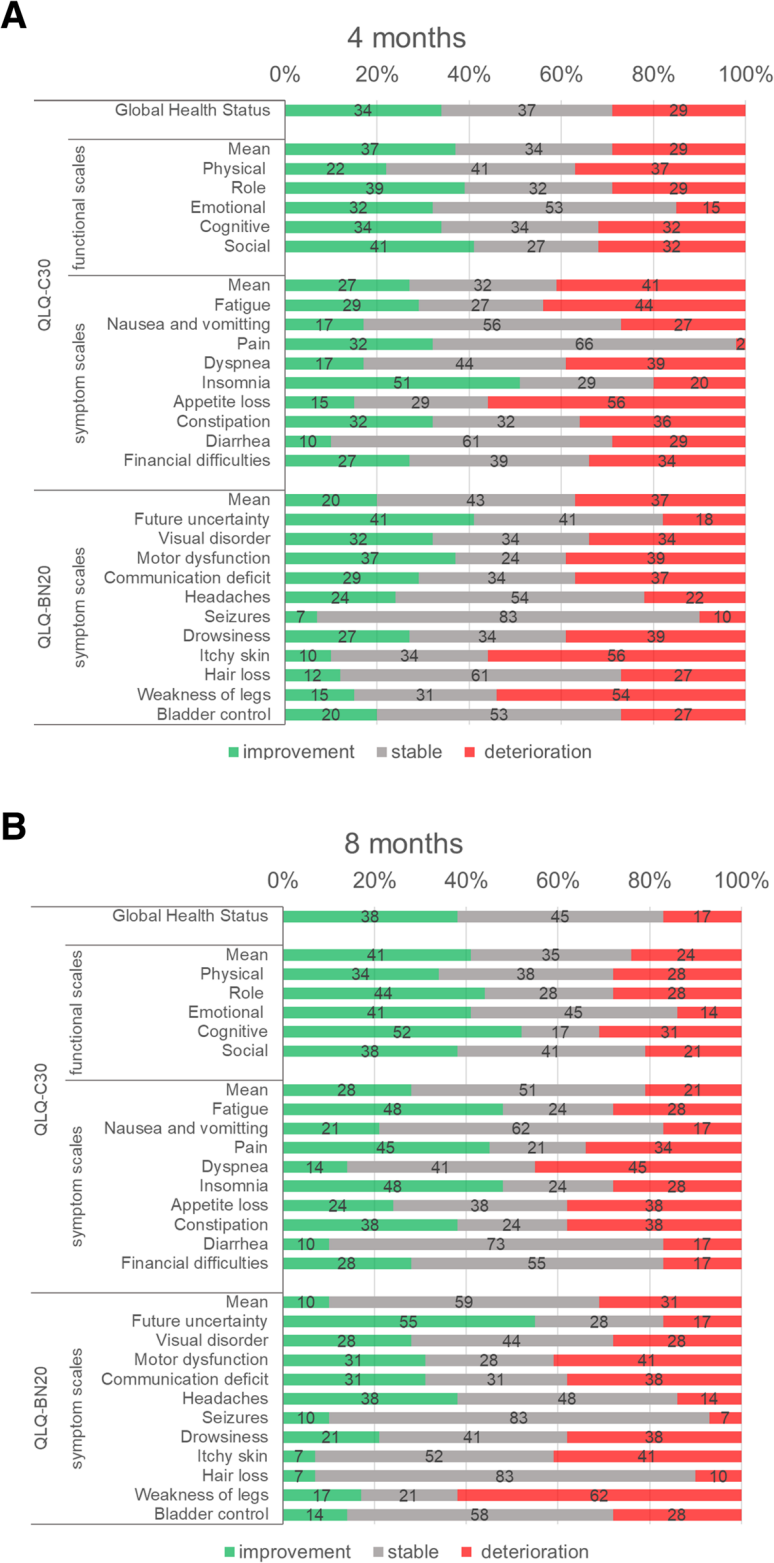
Changes in the patients‘HR-QOL scores from baseline to 4 months (**a**) and from baseline to 8 months (**b**). QLQ-C30: European Organization for Research and Treatment of Cancer (EORTC) Quality of Life Questionnaire Core 30. QLQ-BN20: European Organization for Research and Treatment of Cancer (EORTC) Brain Cancer Module

Regarding the HR-QOL at the scale level, at 4 months (and 8 months), improvement/stable/deterioration was seen 34%/37%/29% (and 38%/45%/17%) in the GHS scale, 37%/34%/29% (and 41%/35%/24%) in the mean QLQ-C30 functional scales, 27%/32%/41% (and 28%/51%/21%) in the mean QLQ-C30 symptom scales, and 20%/43%/37% (and 10%/59%/31%) in the mean QLQ-BN20 scales (Fig. 2).

#### The relationship between the changes in NCF and HR-QOL at 4 months

The scales on which the ‘NCF-deterioration group’ was significantly worse than the other statuses were physical function with 2.0 SD as the cut-off, and social function, nausea and vomiting, and visual disorder with 1.0 SD. The scales on which the ‘NCF-improvement group’ was significantly better than the other statuses were social function and future uncertainty with 1.5 SD (Additional file 2: Table S2). Regarding the mean QLQ-C30/BN-20 scales, the mean QLQ-C30 functional scale score was significantly worse in the NCF-deterioration group than the other statuses with 1.0 SD (*p* = 0.013) and 1.5 SD (*p* = 0.015), but not significantly different with 2.0 SD. The mean QLQ-BN20 scale score was significantly better in the NCF-improvement group than the others only with 1.5 SD (*p* = 0.033). However, when ‘both’ was included in either ‘deterioration’ or ‘improvement,’ no significant difference in HR-QOL was detected (Table 3).

**Table 3:** Number of patients with ≥10-point score deterioration and improvement in HR-QOL from baseline in each category of NCF by using three cut-off values at 4 months

	n (%: n/No. of patient category)				*p*-value	*p*-value
DETERIORATION	Improvement	Stable	Deterioration	Both	Deterioration vs. others	Deterioration/Both vs. others
QLQ-C30:						
Global Health Status						
1.0 SD	2 (33)	2 (40)	4 (24)	4 (31)	0.729	0.701
1.5 SD	2 (25)	4 (44)	3 (20)	3 (33)	0.480	0.507
2.0 SD	1 (14)	6 (40)	3 (20)	2 (50)	0.480	0.744
Functional scales						
1.0 SD	1 (17)	1 (20)	9 (53)	1 (8)	0.013*	0.457
1.5 SD	1 (13)	2 (22)	8 (53)	1 (11)	0.015*	0.296
2.0 SD	1 (14)	3 (20)	7 (47)	1 (25)	0.083	0.168
Symptom scales						
1.0 SD	2 (33)	1 (20)	8 (47)	4 (31)	0.328	0.716
1.5 SD	2 (25)	3 (33)	7 (47)	3 (33)	0.336	0.519
2.0 SD	2 (29)	6 (40)	6 (40)	1 (25)	0.749	1.000
QLQ-BN20:						
1.0 SD	2 (33)	0 (0)	8 (47)	5 (39)	0.328	0.168
1.5 SD	2 (25)	2 (22)	7 (47)	4 (44)	0.336	0.195
2.0 SD	2 (29)	4 (27)	7 (47)	2 (50)	0.336	0.211
IMPROVEMENT	Improvement	Stable	Deterioration	Both	Improvement vs. others	Improvement/Both vs. others
QLQ-C30:						
Global Health Status						
1.0 SD	3 (50)	1 (20)	1 (20)	5 (39)	0.393	0.346
1.5 SD	3 (38)	2 (22)	2 (22)	5 (56)	1.000	0.189
2.0 SD	3 (43)	4 (27)	4 (27)	2 (50)	0.673	0.463
Functional scales						
1.0 SD	4 (67)	1 (20)	1 (20)	4 (31)	0.168	0.533
1.5 SD	5 (63)	3 (33)	3 (33)	2 (22)	0.117	0.745
2.0 SD	4 (57)	5 (33)	5 (33)	1 (25)	0.390	0.491
Symptom scales						
1.0 SD	2 (33)	1 (20)	1 (20)	3 (23)	0.651	1.000
1.5 SD	3 (38)	3 (33)	3 (33)	2 (22)	0.658	1.000
2.0 SD	2 (29)	4 (27)	4 (27)	1 (25)	1.000	1.000
QLQ-BN20:						
1.0 SD	3 (50)	1 (20)	1 (20)	1 (8)	0.077	1.000
1.5 SD	4 (50)	1 (11)	1 (11)	0 (0)	0.033*	0.698
2.0 SD	2 (29)	3 (20)	3 (20)	0 (0)	0.606	1.000

*p-value < 0.05

Both significant improvement and deterioration were observed in different NCF domains *HR-QOL* health-related quality of life, *NCF* neurocognitive function. *QLQ-C30* European Organization for Research and Treatment of Cancer (EORTC) Quality of Life Questionnaire Core 30. *QLQ-BN20* European Organization for Research and Treatment of Cancer (EORTC) Brain Cancer Module

#### The factors related to the deterioration of NCF and HR-QOL at 4 months

When the cut-off value of 1.5 SD was used, the significant factors for the deterioration of the patients’ NCF test at 4 months were (1) intracranial tumor control (non-PD vs. PD) in two domains, and (2) the availability of 8-month examination data in four domains. The significant factor for the deterioration of the patients’ HR-QOL at 4 months were the availability of 8-month examination data in the mean QLQ-C30 functional scale scores, and the use of the molecular targeted therapy after WBRT in the mean QLQ-BN20 scores (Table 4).

**Table 4:** Percentage of patients divided into two groups (age, GPA, number of examinations, intracranial control at 4 months, Systematic therapies prior WBRT, Cytotoxic chemotherapy after WBRT, and Molecular targeted therapy after WBRT) with deterioration in NCF scores with 1.5 SD and HR-QOL scores at 4 months

Factors	Age group, %		p	GPA, %		p	No. of examinations, %		p	Intracranial control at 4 mos., %		p
	< 65	≥65		4.0–2.0	1.5–0.0		3	2		Non-PD	PD	
NCF:												
TR	33	15	0.28	24	25	1.00	14	50	0.04*	8	62	< 0.01*
DR	38	15	0.16	18	33	0.31	14	58	< 0.01*	15	46	0.06
DRec	29	30	1.00	29	29	1.00	17	58	0.02*	23	39	0.45
COWAT	14	10	1.00	24	4	0.14	10	17	0.62	8	23	0.31
TMT-A	24	35	0.51	29	29	1.00	21	50	0.13	23	46	0.16
TMT-B	19	25	0.72	18	25	0.71	10	50	0.01*	4	54	< 0.01*
HR-QOL:												
QLQ-C30;												
Global Health Status	29	30	1.00	29	29	1.00	31	25	1.00	35	15	0.28
Functional scales	29	30	1.00	18	38	0.30	17	58	0.02*	19	46	0.13
Symptom scales	43	30	0.52	41	33	0.75	35	42	0.73	35	39	1.00
QLQ-BN20	33	40	0.75	41	33	0.75	35	42	0.73	35	31	1.00
Factors	Systemic therapies prior to WBRT, %			p	Cytotoxic chemotherapy after WBRT, %			p	Molecular targeted therapy after WBRT, %			p
	No	Yes			No	Yes			No	Yes		
NCF:												
TR	13	32		0.27	27	21		0.73	18	32		0.47
DR	19	32		0.48	27	26		1.00	23	32		0.73
DRec	25	32		0.73	23	37		0.49	27	32		1.00
COWAT	13	12		1.00	14	11		1.00	18	5		0.35
TMT-A	31	28		1.00	18	42		0.17	36	21		0.33
TMT-B	6	32		0.07	23	21		1.00	18	26		0.71
HR-QOL:												
QLQ-C30;												
Global Health Status	31	28		1.00	36	21		0.33	36	21		0.33
Functional scales	19	36		0.31	23	37		0.49	32	26		0.74
Symptom scales	38	36		1.00	36	37		1.00	46	26		0.33
QLQ-BN20	38	36		1.00	32	42		0.53	55	16		0.02*

*p-value < 0.05

*NCF* neurocognitive function, *TR* Totall Recall domain of the Hopkins Verbal Learning Test-Revised (HVLT-R), *DR* Delayed Recall domain of the HVLT-R, *DRec* the Delayed Recognition domain of the HVLT-R, *COWAT* the Control Oral Word Association Test, *TMT-A* part A of the Trail-Making Test (TMT), *TMT-B* part B of the TMT, *HR-QOL* health-related quality of life, *GPA* Graded Prognosis Assessment, *PD* progressive disease. *QLQ-C30* European Organization for Research and Treatment of Cancer (EORTC) Quality of Life Questionnaire Core 30. *QLQ-BN20* European Organization for Research and Treatment of Cancer (EORTC) Brain Cancer Module

## Discussion

The avoidance of WBRT is a current trend in some clinical situations [15], but WBRT is still important for most cases of brain metastases [16]. Recent prospective studies demonstrated that the use of WBRT is more frequently associated with cognitive side effects compared to stereotactic radiosurgery (SRS) alone. However, the role of WBRT is to improve the intracranial tumor control, and there is possibility that NCF deteriorate due to progression intracranial tumor by omitting WBRT [2]. In the JROSG 99–1 Study by Aoyama et al., the patients assigned to WBRT+SRS group demonstrated better short-term NCF compared with those assigned to SRS alone group. On the other hand, in this study, intracranial tumor control has been shown to be related to NCF deterioration at 4 months. Moreover, the reported percentage of patients suffering neurocognitive deterioration varies widely among the publications because of the lack of standardization in the NCF score change criteria. In the Alliance trial comparing WBRT+SRS and SRS-alone, cognitive deterioration was defined as deterioration over 1.0 SD from baseline on at least one domain at 3 months post-WBRT. That trial’s authors Brown PD et al. reported that cognitive deterioration was seen in 91.7% of the patients in the WBRT+SRS arm and 63.5% in the SRS-alone arm (*p* < 0.001) [6]. In the MD Anderson Cancer Center trial in the same setting, 52% of the patients in the WBRT+SRS arm and 24% in the SRS-alone arm showed neurocognitive deterioration at 24 weeks after treatment [7]. In that trial, a 5-point drop in any domain of the HVLT-R was defined as deterioration. In the RTOG 0614 study comparing WBRT with or without memantine, the cut off value for cognitive failure were 2SD and the reliable change index [17]. The probability of cognitive function failure at 24 weeks was 53.8% in the memantine arm and 64.9% in the placebo arm (*p* = 0.01) [8].

There are the two pitfalls in interpreting these data. One is that some patients in the deterioration group could have both deterioration in one or more domains and improvement in other domains. These ‘both’ patients could be included in an improvement group if the ‘improvement on at least one domain’ definition was applied. In a study that assessed the changes in NCF after SRS alone, van der Meer et al. used the cut-off of 1.5 SD and categorized their patients into four groups: improvement (14%), stable (67%), deterioration (14%), and both (6%) at 3 months after the initial SRS [11]. In the present study, as much as 32% of the patients (with 1.0 SD as the cut-off), 22% (with 1.5 SD), or 9% (with 2.0 SD) showed both deterioration and improvement at 4 months. When these ‘both’ patients are included in the deterioration group or the improvement group with the cut-off of 1.0 SD, the percentage of patients with deterioration and the percentage of those with improvement increase from 41 to 73% (41% + 32%) and from 15 to 47% (15% + 32%), respectively.

The other pitfall in the data interpretation is that the cut-off value is different for each study. Therefore, we tried to construct the NCF change criteria based on HR-QOL because the goal of the treatment of brain metastases is the maintenance or even improvement of the patient’s HR-QOL, considering the palliative nature of the treatment. In addition, it is known that NCF and HR-QOL are related after WBRT for brain metastasis [18]. It should be noted that assessments of ‘clinically meaningful changes in HR-QOL status’ is another topic for research, but we used the HR-QOL cut-off value of ≥10 points, which reflects a moderate change in the HR-QOL status in the QLQ-C30 and the QLQ-BN20 [19, 20]. In the present analyses, based on the patients’ mean QLQ-C30 functional scale scores with 1.0 SD and 1.5SD, a clinically meaningful deterioration of the patients’ HR-QOL was observed significantly more frequently in the NCF deterioration group compared to the NCF improvement group, stable group, and both-deterioration-and-improvement groups at 4 months.

On the other hand, a clinically meaningful improvement of the patients’ HR-QOL was observed significantly more frequently in the NCF improvement group compared to the other three patient groups based on the mean of the QLQ-BN20 scores with 1.5 SD at 4 months. However, when the ‘both’ patients were included in the deterioration group or the improvement group, the statistical significance of all of the above findings was lost. This implies that patients who show both deterioration and improvement in different domains should be differentiated from patients who exhibit only deterioration or only improvement. Therefore, the use of ‘at least one domain’ for the definition of deterioration/improvement should be interpreted carefully, and the automatic inclusion of these ‘both’ patients in a deterioration group should be questioned.

Our present findings demonstrate that the use of a stricter cut-off value (i.e., 2.0 SD) resulted in a reduction of the number of ‘both’ patients but provided less sensitivity, and it could not detect significant HR-QOL changes. We thus propose that the cut-off value of 1.5 SD and the exclusion of ‘both’ patients from the deterioration and improvement groups would most closely reflect the clinically meaningful changes in HR-QOL.

Our study has several limitations. First, although the examination compliance in our study population was better (85% at 4 months) than that reported in other studies (59–73% at 2–4 months) [6, 8, 11, 21–23], there were patients who could not perform the follow up examinations. Second, this study was unable to perform multivariate analysis that considers confounding factors related to cognitive dysfunction such as opioids [24], chemotherapy [25], surgery, etc.. However, in this study patients group, it has been confirmed that the presence or absence of surgery/chemotherapy did not cause a significant difference in NCF tests at baseline. Third, the prognoses of our study population were heterogeneous, since patients who have undergone WBRT postoperatively and patients with meningeal carcinomatosis were analyzed together. The prognosis of individuals with brain metastases has been prolonged, and it may be necessary to change the time points of the NCF and HR-QOL examinations according to patients’ prognoses. Finally, due to the small sample size, we could not fully analyze the patients’ NCF and HR-QOL after 8 months post-WBRT. It is necessary to verify our present findings with a larger number of patients over longer follow-up periods.

## Conclusions

The use of 1.5 SD as the cut-off for NCF best reflected the patients’ HR-QOL status. The inclusion of ‘both’ in either a ‘deterioration’ or ‘improvement’ group blurs the changes in NCF reflecting the clinical meaningful changes in HR-QOL, and therefore patients showing both deterioration and improvement should not be included in either group.

## Supplementary information

**Additional file 1: Figure S1.** Overall survival in the patients who underwent examinations at baseline only, at baseline and 4 months only, or at baseline, 4 months and 8 months. BL: baseline. MST: median survival time. WBRT: whole-brain radiotherapy.

**Additional file 2: Table S1.** The mean scores in examinations at baseline and comparisons of pairs of groups. **Table S2.** Including full scales/items, Number of patients with ≥10 score deterioration and improvement in HR-QOL from BL in each category of NCF by using three cut-off values at 4 months.

## Data Availability

The datasets generated during and/or analysed during the current study are available from the corresponding author on reasonable request.

## Abbreviations

COWAT: Control Oral Word Association Test
DR: Delayed Recall
DRec: Delayed Recognition
EORTC: European Organization for Research and Treatment of Cancer
GHS: global health status
GPA: Graded Prognostic Assessment
HR-QOL: Health-related quality of life
HVLT-R: Hopkins Verbal Learning Test-Revised
KPS: Karnofsky Performance Status
MST: median survival time
NCF: Neurocognitive function
PD: progressive disease
QLQ-BN20: Quality of Life Questionnaire Brain Cancer Module
QLQ-C30: Quality of Life Questionnaire Core 30
RANO: Response Assessment in Neuro-Oncology
SDs: standard deviations
SRS: stereotactic radiosurgery
TMT: Trail-Making Test
TMT-A: Parts A of the TMT
TMT-B: Parts B of the TMT
TR: Total Recall
WBRT: whole-brain radiation therapy
