# Supplementary material for: The neurocognitive function change criteria after whole-brain radiation therapy for brain metastasis, in reference to health-related quality of life changes: a prospective observation study
Source: BMC Cancer. 2020 Jan 29;20:66. doi: 10.1186/s12885-020-6559-3 (PMC6988195; doi:10.1186/s12885-020-6559-3)
Supplement: Supplementary file 1 — Additional file 1: Figure S1. Overall survival in the patients who underwent examinations at baseline only, at baseline and 4 months only, or at baseline, 4 months and 8 months. BL: baseline. MST: median survival time. WBRT: whole-brain radiotherapy. [file 12885_2020_6559_MOESM1_ESM.pptx]

## Slide 1
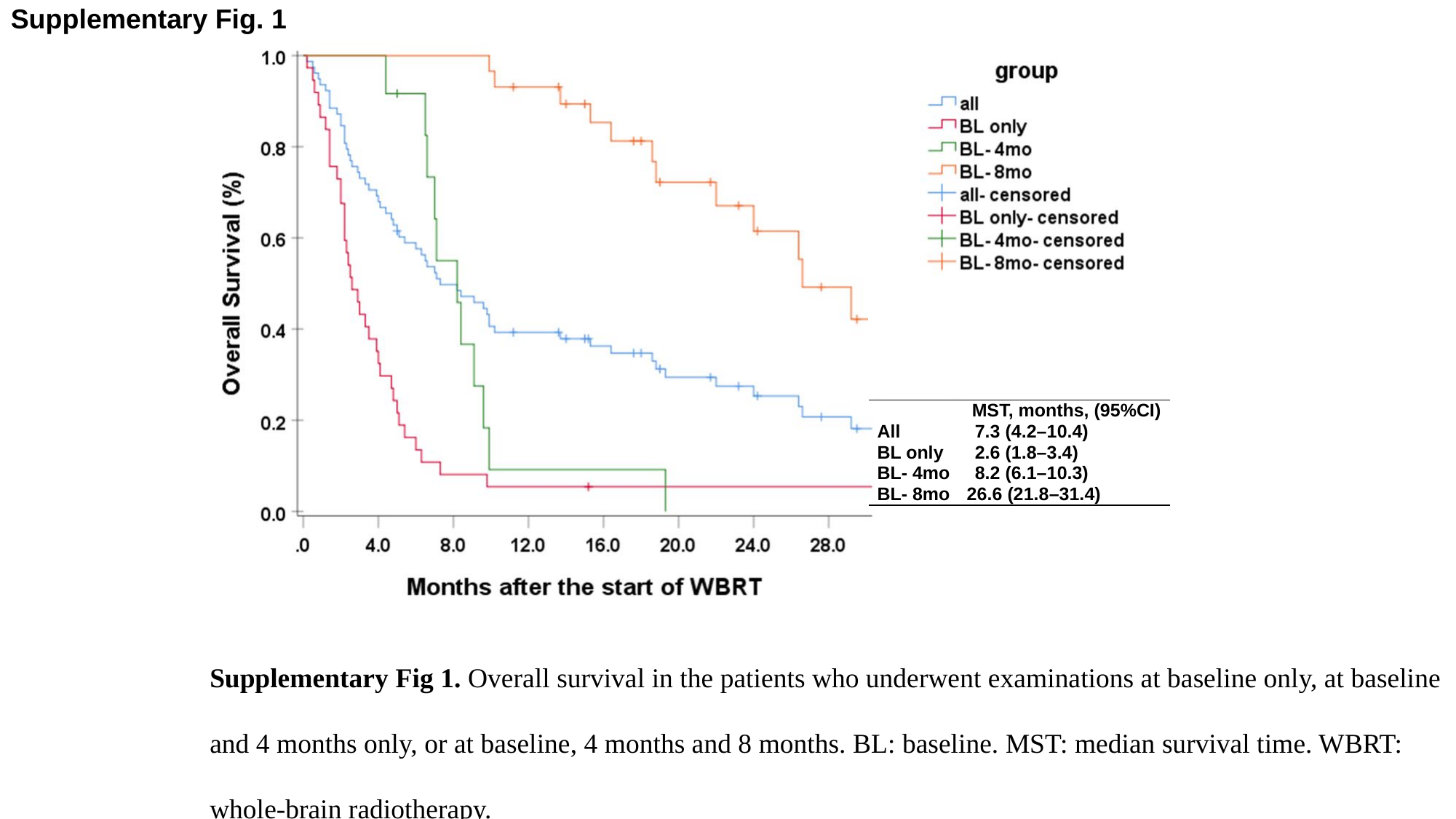

Supplementary Fig. 1
| | MST, months, (95%CI) |
| --- | --- |
| All | 7.3 (4.2–10.4) |
| BL only | 2.6 (1.8–3.4) |
| BL- 4mo | 8.2 (6.1–10.3) |
| BL- 8mo | 26.6 (21.8–31.4) |
Supplementary Fig 1. Overall survival in the patients who underwent examinations at baseline only, at baseline and 4 months only, or at baseline, 4 months and 8 months. BL: baseline. MST: median survival time. WBRT: whole-brain radiotherapy.
