# Supplementary material for: The neurocognitive function change criteria after whole-brain radiation therapy for brain metastasis, in reference to health-related quality of life changes: a prospective observation study
Source: BMC Cancer. 2020 Jan 29;20:66. doi: 10.1186/s12885-020-6559-3 (PMC6988195; doi:10.1186/s12885-020-6559-3)
Supplement: Supplementary file 2 — Additional file 2: Table S1. The mean scores in examinations at baseline and comparisons of pairs of groups. Table S2. Including full scales/items, Number of patients with ≥10 score deterioration and improvement in HR-QOL from BL in each category of NCF by using three cut-off values at 4 months. [file 12885_2020_6559_MOESM2_ESM.docx]

**Supplementary Table 1.** The mean scores in examinations at baseline and comparisons of pairs of groups

**1-a. All, Age, Karnofsky Performance Status (KPS)**

| **Factors** | **All** | **Age** | | | | | | **KPS** | | | | | |  |
| --- | --- | --- | --- | --- | --- | --- | --- | --- | --- | --- | --- | --- | --- | --- |
|  |  | | **<65** | | **≥65** | | **p** | | **100–80** | | **70–60** | | **p** | |
| **NCF:** |  |  | |  | |  | |  | |  | |  | |  |
| TR | −1.46 | −1.32 | | −1.66 | | 0.23 | | −1.26 | | −1.82 | | 0.18 | |  |
| DR | −1.75 | −1.69 | | −1.83 | | 0.59 | | −1.60 | | −2.03 | | 0.38 | |  |
| DRec | −1.07 | −1.00 | | −1.17 | | 0.17 | | −0.69 | | −1.76 | | 0.08 | |  |
| COWAT | −0.46 | −0.52 | | −0.40 | | 0.67 | | −0.21 | | −0.98 | | **0.01** | |  |
| TMT-A | −1.46 | −1.38 | | −1.58 | | 0.52 | | −1.27 | | −1.88 | | 0.30 | |  |
| TMT-B | −1.12 | −1.11 | | −1.13 | | 0.56 | | −0.91 | | −1.54 | | **0.01** | |  |
| **HR-QOL:** |  |  | |  | |  | |  | |  | |  | |  |
| **QLQ-C30:** |  |  | |  | |  | |  | |  | |  | |  |
| Global health status | 41.9 | 41.3 | | 42.7 | | 0.53 | | 47.3 | | 32.1 | | **0.01** | |  |
| **Functional scales:** |  |  | |  | |  | |  | |  | |  | |  |
| Physical | 63.9 | 65.8 | | 61.4 | | 0.29 | | 74.0 | | 46.0 | | **<0.01** | |  |
| Role | 51.9 | 53.3 | | 50.0 | | 0.67 | | 60.7 | | 36.3 | | **<0.01** | |  |
| Emotional | 70.1 | 69.4 | | 71.0 | | 0.74 | | 73.7 | | 63.7 | | **0.03** | |  |
| Cognitive | 60.0 | 64.1 | | 54.5 | | **0.03** | | 67.0 | | 47.6 | | **<0.01** | |  |
| Social | 66.2 | 61.5 | | 72.7 | | 0.07 | | 74.3 | | 51.8 | | **<0.01** | |  |
| **Symptom scales:** |  |  | |  | |  | |  | |  | |  | |  |
| Fatigue | 43.0 | 42.5 | | 43.8 | | 0.73 | | 37.3 | | 53.2 | | **0.01** | |  |
| Nausea/vomiting | 9.2 | 11.1 | | 6.6 | | 0.39 | | 6.3 | | 14.3 | | 0.11 | |  |
| Pain | 30.8 | 27.8 | | 34.8 | | 0.22 | | 25.3 | | 40.5 | | 0.09 | |  |
| Dyspnea | 29.1 | 26.7 | | 32.3 | | 0.48 | | 20.7 | | 44.0 | | **<0.01** | |  |
| Insomnia | 37.6 | 36.3 | | 39.4 | | 0.58 | | 28.0 | | 54.8 | | **<0.01** | |  |
| Appetite loss | 32.1 | 34.1 | | 29.3 | | 0.34 | | 27.3 | | 40.5 | | 0.12 | |  |
| Constipation | 34.6 | 31.9 | | 38.4 | | 0.45 | | 30.7 | | 41.7 | | 0.20 | |  |
| Diarrhea | 12.4 | 13.3 | | 11.1 | | 0.96 | | 11.3 | | 14.3 | | 0.73 | |  |
| Financial difficulties | 32.5 | 36.3 | | 27.3 | | 0.19 | | 25.3 | | 45.2 | | **<0.01** | |  |
| **QLQ-BN20:** |  |  | |  | |  | |  | |  | |  | |  |
| Future uncertainty | 45.7 | 45.9 | | 45.5 | | 0.98 | | 40.3 | | 55.4 | | **0.01** | |  |
| Visual disorder | 20.9 | 16.2 | | 27.3 | | **0.03** | | 17.1 | | 28.0 | | **0.03** | |  |
| Motor dysfunction | 21.1 | 16.2 | | 27.6 | | **0.01** | | 14.0 | | 34.2 | | **<0.01** | |  |
| Communication deficit | 19.2 | 11.4 | | 29.6 | | **<0.01** | | 13.1 | | 30.5 | | **<0.01** | |  |
| Headaches | 22.1 | 19.7 | | 25.3 | | 0.50 | | 22.0 | | 22.2 | | 0.70 | |  |
| Seizures | 5.6 | 4.5 | | 7.1 | | 0.44 | | 2.7 | | 11.1 | | 0.18 | |  |
| Drowsiness | 37.2 | 33.3 | | 42.4 | | 0.19 | | 30.0 | | 50.6 | | **0.01** | |  |
| Itchy skin | 12.1 | 9.1 | | 16.2 | | 0.31 | | 8.7 | | 18.5 | | 0.06 | |  |
| Hair loss | 13.4 | 9.1 | | 19.2 | | 0.20 | | 10.0 | | 19.8 | | 0.11 | |  |
| Weakness of legs | 43.3 | 40.9 | | 46.5 | | 0.34 | | 35.3 | | 58.0 | | **<0.01** | |  |
| Bladder control | 17.7 | 7.6 | | 31.3 | | **<0.01** | | 10.0 | | 32.1 | | **<0.01** | |  |

**1-b. No. of brain metastases, Surgery for brain metastases (BMs)**

| **Factors** | **No. of BMs** | | | | | **Surgery for BMs** | | | | | |  |
| --- | --- | --- | --- | --- | --- | --- | --- | --- | --- | --- | --- | --- |
|  | **1 to 4** | | **≥5 or meningeal carcinomatosis** | | **p** | | **No** | | **Yes** | | **p** | |
| **NCF:** |  |  | |  | |  | |  | |  | |  |
| TR | −1.63 | −1.34 | | 0.41 | | −1.46 | | −1.48 | | 0.78 | |  |
| DR | −2.06 | −1.53 | | 0.27 | | −1.66 | | −2.01 | | 0.46 | |  |
| DRec | −1.05 | −1.09 | | 0.97 | | −1.16 | | −0.80 | | 0.66 | |  |
| COWAT | −0.70 | −0.29 | | 0.31 | | −0.42 | | −0.59 | | 0.33 | |  |
| TMT-A | −1.77 | −1.27 | | 0.56 | | −1.30 | | −1.95 | | 0.26 | |  |
| TMT-B | −1.42 | −0.94 | | 0.48 | | −0.95 | | −1.66 | | 0.14 | |  |
| **HR-QOL:** |  |  | |  | |  | |  | |  | |  |
| **QLQ-C30** |  |  | |  | |  | |  | |  | |  |
| Global health status | 46.0 | 38.9 | | 0.16 | | 41.7 | | 42.5 | | 0.95 | |  |
| **Functional scales:** |  |  | |  | |  | |  | |  | |  |
| Physical | 65.5 | 62.8 | | 0.55 | | 64.8 | | 61.3 | | 0.43 | |  |
| Role | 47.0 | 55.6 | | 0.33 | | 57.8 | | 35.0 | | **0.01** | |  |
| Emotional | 67.9 | 71.7 | | 0.47 | | 72.6 | | 62.9 | | **<0.05** | |  |
| Cognitive | 54.5 | 64.1 | | **<0.05** | | 62.4 | | 53.3 | | **0.04** | |  |
| Social | 64.6 | 67.4 | | 0.74 | | 67.2 | | 63.3 | | 0.58 | |  |
| **Symptom scales:** |  |  | |  | |  | |  | |  | |  |
| Fatigue | 38.4 | 46.4 | | 0.16 | | 42.9 | | 43.3 | | 0.78 | |  |
| Nausea/vomiting | 8.6 | 9.6 | | 0.81 | | 10.6 | | 5.0 | | 0.19 | |  |
| Pain | 30.3 | 31.1 | | 0.84 | | 31.9 | | 27.5 | | 0.61 | |  |
| Dyspnea | 19.2 | 36.3 | | **0.01** | | 31.0 | | 23.3 | | 0.22 | |  |
| Insomnia | 43.4 | 33.3 | | 0.25 | | 32.2 | | 53.3 | | 0.05 | |  |
| Appetite loss | 34.3 | 30.4 | | 0.68 | | 32.2 | | 31.7 | | 0.83 | |  |
| Constipation | 39.4 | 31.1 | | 0.39 | | 29.9 | | 48.3 | | 0.05 | |  |
| Diarrhea | 11.1 | 13.3 | | 0.76 | | 12.6 | | 11.7 | | 0.82 | |  |
| Financial difficulties | 31.3 | 33.3 | | 0.73 | | 30.5 | | 38.3 | | 0.67 | |  |
| **QLQ-BN20:** |  |  | |  | |  | |  | |  | |  |
| Future uncertainty | 46.2 | 45.4 | | 0.65 | | 44.7 | | 48.8 | | 0.52 | |  |
| Visual disorder | 19.4 | 22.0 | | 0.56 | | 20.7 | | 21.7 | | 0.82 | |  |
| Motor dysfunction | 24.0 | 19.0 | | 0.50 | | 18.1 | | 29.4 | | 0.12 | |  |
| Communication deficit | 27.1 | 13.6 | | 0.12 | | 17.7 | | 23.3 | | 0.35 | |  |
| Headaches | 22.9 | 21.5 | | 0.94 | | 23.4 | | 18.3 | | 0.52 | |  |
| Seizures | 6.3 | 5.2 | | 0.93 | | 4.7 | | 8.3 | | 0.82 | |  |
| Drowsiness | 37.5 | 37.0 | | 0.92 | | 35.7 | | 41.7 | | 0.51 | |  |
| Itchy skin | 6.3 | 16.3 | | **0.04** | | 13.5 | | 8.3 | | 0.18 | |  |
| Hair loss | 8.3 | 17.0 | | 0.07 | | 14.0 | | 11.7 | | 0.36 | |  |
| Weakness of legs | 42.7 | 43.7 | | 0.89 | | 41.5 | | 48.3 | | 0.39 | |  |
| Bladder control | 17.7 | 17.8 | | 0.72 | | 17.0 | | 20.0 | | 0.86 | |  |

**1-c. Number of examinations, Graded Prognostic Assessment (GPA)**

| **Factors** | **No. of examinations** | | | | | **GPA** | | | | | |  |
| --- | --- | --- | --- | --- | --- | --- | --- | --- | --- | --- | --- | --- |
|  | **≥2** | | **1** | | **p** | | **4.0–2.0** | | **1.5–0.0** | | **p** | |
| **NCF:** |  |  | |  | |  | |  | |  | |  |
| TR | −1.40 | −1.53 | | 0.93 | | −1.34 | | −1.52 | | 0.61 | |  |
| DR | −1.66 | −1.85 | | 0.62 | | −1.80 | | −1.73 | | 0.91 | |  |
| DRec | −0.67 | −1.52 | | 0.49 | | −0.80 | | −1.20 | | 0.44 | |  |
| COWAT | −0.30 | −0.66 | | 0.47 | | −0.49 | | −0.45 | | 0.97 | |  |
| TMT-A | −1.61 | −1.29 | | 0.87 | | −1.64 | | −1.38 | | 0.83 | |  |
| TMT-B | −0.79 | −1.44 | | 0.06 | | −1.26 | | −1.06 | | 0.72 | |  |
| **HR-QOL:** |  |  | |  | |  | |  | |  | |  |
| **QLQ-C30** |  |  | |  | |  | |  | |  | |  |
| Global health status | 45.9 | 37.4 | | 0.16 | | 50.3 | | 37.9 | | **0.04** | |  |
| **Functional scales:** |  |  | |  | |  | |  | |  | |  |
| Physical | 66.5 | 61.1 | | 0.41 | | 76.5 | | 58.0 | | **<0.01** | |  |
| Role | 50.8 | 53.2 | | 0.79 | | 56.7 | | 49.7 | | 0.40 | |  |
| Emotional | 71.3 | 68.7 | | 0.66 | | 74.3 | | 68.1 | | 0.33 | |  |
| Cognitive | 58.1 | 62.2 | | 0.41 | | 63.3 | | 58.5 | | 0.54 | |  |
| Social | 70.7 | 61.3 | | 0.11 | | 70.7 | | 64.2 | | 0.42 | |  |
| **Symptom scales:** |  |  | |  | |  | |  | |  | |  |
| Fatigue | 42.3 | 43.8 | | 0.86 | | 35.1 | | 46.8 | | **0.03** | |  |
| Nausea/vomiting | 6.9 | 11.7 | | 0.22 | | 2.7 | | 12.3 | | **<0.01** | |  |
| Pain | 28.9 | 32.9 | | 0.63 | | 18.7 | | 36.5 | | **0.01** | |  |
| Dyspnea | 22.8 | 36.0 | | **<0.05** | | 16.0 | | 35.2 | | **<0.01** | |  |
| Insomnia | 36.6 | 38.7 | | 0.62 | | 33.3 | | 39.6 | | 0.35 | |  |
| Appetite loss | 27.6 | 36.9 | | 0.17 | | 25.3 | | 35.2 | | 0.21 | |  |
| Constipation | 29.3 | 40.5 | | 0.09 | | 30.7 | | 36.5 | | 0.45 | |  |
| Diarrhea | 5.7 | 19.8 | | **0.02** | | 10.7 | | 13.2 | | 0.99 | |  |
| Financial difficulties | 30.9 | 34.2 | | 0.68 | | 24.0 | | 36.5 | | 0.06 | |  |
| **QLQ-BN20:** |  |  | |  | |  | |  | |  | |  |
| Future uncertainty | 43.3 | 48.4 | | 0.51 | | 37.7 | | 49.5 | | 0.06 | |  |
| Visual disorder | 20.3 | 21.6 | | 0.79 | | 12.0 | | 25.2 | | **0.04** | |  |
| Motor dysfunction | 19.8 | 22.5 | | 0.51 | | 13.8 | | 24.6 | | **0.03** | |  |
| Communication deficit | 22.0 | 16.0 | | 0.37 | | 15.6 | | 20.9 | | 0.17 | |  |
| Headaches | 25.2 | 18.5 | | 0.42 | | 18.7 | | 23.7 | | 0.55 | |  |
| Seizures | 5.7 | 5.6 | | 0.62 | | 1.3 | | 7.7 | | 0.27 | |  |
| Drowsiness | 34.1 | 40.7 | | 0.38 | | 30.7 | | 40.4 | | 0.26 | |  |
| Itchy skin | 12.2 | 12.0 | | 0.79 | | 5.3 | | 15.4 | | **0.03** | |  |
| Hair loss | 9.8 | 17.6 | | **0.02** | | 4.0 | | 17.9 | | **0.02** | |  |
| Weakness of legs | 36.6 | 50.9 | | 0.07 | | 30.7 | | 49.4 | | **0.01** | |  |
| Bladder control | 16.3 | 19.4 | | 0.38 | | 8.0 | | 22.4 | | 0.08 | |  |

**1-d. Systematic therapies prior to WBRT**

| **Factors** | **Systematic therapies**  **Prior to WBRT** | | | | |  |
| --- | --- | --- | --- | --- | --- | --- |
|  | **No** | | **Yes** | | **p** | |
| **NCF:** |  |  | |  | |  |
| TR | -1.78 | -1.33 | | 0.22 | |  |
| DR | -2.07 | -1.62 | | 0.22 | |  |
| DRec | -1.26 | -0.99 | | 0.28 | |  |
| COWAT | -0.30 | -0.53 | | 0.32 | |  |
| TMT-A | -1.65 | -1.39 | | 1.00 | |  |
| TMT-B | -0.62 | -1.27 | | 0.39 | |  |
| **HR-QOL:** |  |  | |  | |  |
| **QLQ-C30** |  |  | |  | |  |
| Global health status | 47.5 | 39.5 | | 0.22 | |  |
| **Functional scales:** |  |  | |  | |  |
| Physical | 60.3 | 65.5 | | 0.39 | |  |
| Role | 41.3 | 56.4 | | 0.11 | |  |
| Emotional | 68.1 | 70.9 | | 0.43 | |  |
| Cognitive | 58.7 | 60.6 | | 0.49 | |  |
| Social | 68.8 | 65.2 | | 0.62 | |  |
| **Symptom scales:** |  |  | |  | |  |
| Fatigue | 40.1 | 44.2 | | 0.50 | |  |
| Nausea/vomiting | 4.3 | 11.2 | | **0.04** | |  |
| Pain | 32.6 | 30.0 | | 0.89 | |  |
| Dyspnea | 18.8 | 33.3 | | **0.02** | |  |
| Insomnia | 47.8 | 33.3 | | 0.14 | |  |
| Appetite loss | 24.6 | 35.2 | | 0.13 | |  |
| Constipation | 36.2 | 33.9 | | 0.81 | |  |
| Diarrhea | 4.3 | 15.8 | | 0.06 | |  |
| Financial difficulties | 27.5 | 34.5 | | 0.32 | |  |
| **QLQ-BN20:** |  |  | |  | |  |
| Future uncertainty | 42.4 | 47.1 | | 0.51 | |  |
| Visual disorder | 17.9 | 22.2 | | 0.33 | |  |
| Motor dysfunction | 20.3 | 21.4 | | 0.86 | |  |
| Communication deficit | 26.6 | 16.0 | | 0.11 | |  |
| Headaches | 26.1 | 20.4 | | 0.49 | |  |
| Seizures | 7.2 | 4.9 | | 0.45 | |  |
| Drowsiness | 36.2 | 37.7 | | 0.99 | |  |
| Itchy skin | 8.7 | 13.6 | | 0.38 | |  |
| Hair loss | 2.9 | 17.9 | | **<0.01** | |  |
| Weakness of legs | 40.6 | 44.4 | | 0.75 | |  |
| Bladder control | 17.4 | 17.9 | | 0.97 | |  |

NCF: neurocognitive function. HR-QOL: health-related quality of life. QLQ-C30: European Organization for Research and Treatment of Cancer (EORTC) Quality of Life Questionnaire Core 30. QLQ-BN20: European Organization for Research and Treatment of Cancer (EORTC) Brain Cancer Module.

**Supplementary table 2.** Including full scales/items, Number of patients with ≥ 10 score deterioration and improvement in HR-QOL from BL in each category of NCF by using three cut-off values at 4 months

|  | **N (%: N/No. of patient category)** | | | | p | p |
| --- | --- | --- | --- | --- | --- | --- |
| **Deterioration** | **Improvement** | **Stable** | **Deterioration** | **Both** | **Deterioration vs. others** | **Deterioration/Both vs. others** |
| **QLQ-C30** |  |  |  |  |  |  |
| **Global health status** |  |  |  |  |  |  |
| 1.0 SD | 2 (33) | 2 (40) | 4 (24) | 4 (31) | 0.729 | 0.701 |
| 1.5 SD | 2 (25) | 4 (44) | 3 (20) | 3 (33) | 0.480 | 0.507 |
| 2.0 SD | 1 (14) | 6 (40) | 3 (20) | 2 (50) | 0.480 | 0.744 |
| **Functional scales:** |  |  |  |  |  |  |
| **Physical** |  |  |  |  |  |  |
| 1.0 SD | 2 (33) | 2 (40) | 8 (47) | 3 (23) | 0.328 | 1.000 |
| 1.5 SD | 2 (25) | 2 (22) | 8 (53) | 3 (33) | 0.108 | 0.195 |
| 2.0 SD | 2 (29) | 3 (20) | 9 (60) | 1 (25) | **0.041** | 0.060 |
| **Role** |  |  |  |  |  |  |
| 1.0 SD | 1 (17) | 2 (40) | 7 (41) | 2 (15) | 0.184 | 1.000 |
| 1.5 SD | 1 (13) | 2 (22) | 7 (47) | 2 (22) | 0.083 | 0.296 |
| 2.0 SD | 1 (14) | 3 (20) | 7 (47) | 1 (25) | 0.083 | 0.168 |
| **Emotional** |  |  |  |  |  |  |
| 1.0 SD | 0 (0) | 0 (0) | 3 (18) | 3 (23) | 0.679 | 0.167 |
| 1.5 SD | 1 (13) | 1 (11) | 2 (13) | 2 (22) | 1.000 | 1.000 |
| 2.0 SD | 1 (14) | 2 (13) | 2 (13) | 1 (25) | 1.000 | 1.000 |
| **Cognitive** |  |  |  |  |  |  |
| 1.0 SD | 2 (33) | 1 (20) | 7 (41) | 3 (23) | 0.322 | 1.000 |
| 1.5 SD | 2 (25) | 2 (22) | 6 (40) | 3 (33) | 0.492 | 0.499 |
| 2.0 SD | 2 (29) | 3 (20) | 7 (47) | 1 (25) | 0.168 | 0.313 |
| **Social** |  |  |  |  |  |  |
| 1.0 SD | 0 (0) | 1 (20) | 9 (53) | 3 (23) | **0.020** | 0.127 |
| 1.5 SD | 0 (0) | 3 (33) | 7 (47) | 3 (33) | 0.168 | 0.173 |
| 2.0 SD | 1 (14) | 6 (40) | 5 (33) | 1 (25) | 1.000 | 1.000 |
| **Symptom scales:** |  |  |  |  |  |  |
| **Fatigue** |  |  |  |  |  |  |
| 1.0 SD | 3 (50) | 2 (40) | 8 (47) | 5 (39) | 0.760 | 1.000 |
| 1.5 SD | 4 (50) | 3 (33) | 9 (60) | 2 (22) | 0.191 | 1.000 |
| 2.0 SD | 3 (43) | 5 (33) | 9 (60) | 1 (25) | 0.191 | 0.355 |
| **Nausea/vomiting** |  |  |  |  |  |  |
| 1.0 SD | 0 (0) | 1 (20) | 8 (47) | 2 (15) | **0.029** | 0.233 |
| 1.5 SD | 0 (0) | 3 (33) | 7 (47) | 1 (11) | 0.064 | 0.309 |
| 2.0 SD | 1 (14) | 4 (27) | 6 (40) | 0 (0) | 0.272 | 0.725 |
| **Pain** |  |  |  |  |  |  |
| 1.0 SD | 1 (17) | 1 (20) | 6 (35) | 6 (46) | 1.000 | 0.275 |
| 1.5 SD | 1 (13) | 2 (22) | 6 (40) | 5 (56) | 0.734 | 0.096 |
| 2.0 SD | 2 (29) | 3 (20) | 6 (40) | 3 (75) | 0.734 | 0.115 |
| **Dyspnea** |  |  |  |  |  |  |
| 1.0 SD | 4 (67) | 1 (20) | 7 (41) | 4 (31) | 1.000 | 0.723 |
| 1.5 SD | 4 (50) | 1 (11) | 7 (47) | 4 (44) | 0.517 | 0.344 |
| 2.0 SD | 3 (43) | 2 (13) | 9 (60) | 2 (50) | 0.051 | **0.029** |
| **Insomnia** |  |  |  |  |  |  |
| 1.0 SD | 0 (0) | 0 (0) | 4 (26) | 4 (31) | 0.698 | 0.083 |
| 1.5 SD | 0 (0) | 1 (11) | 5 (33) | 2 (22) | 0.117 | 0.110 |
| 2.0 SD | 0 (0) | 2 (13) | 5 (33) | 1 (25) | 0.117 | 0.115 |
| **Appetite loss** |  |  |  |  |  |  |
| 1.0 SD | 4 (67) | 4 (80) | 12 (71) | 3 (23) | 0.201 | 0.291 |
| 1.5 SD | 4 (50) | 8 (89) | 9 (60) | 2 (22) | 0.754 | 0.201 |
| 2.0 SD | 3 (43) | 12 (80) | 8 (53) | 0 (0) | 1.000 | 0.122 |
| **Constipation** |  |  |  |  |  |  |
| 1.0 SD | 3 (50) | 1 (20) | 7 (41) | 4 (31) | 0.745 | 1.000 |
| 1.5 SD | 3 (38) | 3 (33) | 6 (40) | 3 (33) | 0.749 | 1.000 |
| 2.0 SD | 2 (29) | 5 (33) | 6 (40) | 2 (50) | 0.749 | 0.533 |
| **Diarrhea** |  |  |  |  |  |  |
| 1.0 SD | 1 (17) | 1 (20) | 6 (35) | 4 (31) | 0.507 | 0.457 |
| 1.5 SD | 2 (25) | 3 (33) | 5 (33) | 2 (22) | 0.730 | 1.000 |
| 2.0 SD | 2 (29) | 5 (33) | 5 (33) | 0 (0) | 0.730 | 0.744 |
| **Financial difficulties** |  |  |  |  |  |  |
| 1.0 SD | 2 (33) | 0 (0) | 7 (41) | 5 (39) | 0.512 | 0.275 |
| 1.5 SD | 4 (50) | 1 (11) | 6 (40) | 3 (33) | 0.734 | 0.742 |
| 2.0 SD | 3 (43) | 4 (27) | 5 (33) | 2 (50) | 1.000 | 0.754 |
| **QLQ-BN20:** |  |  |  |  |  |  |
| **Future uncertainty** |  |  |  |  |  |  |
| 1.0 SD | 1 (17) | 0 (0) | 4 (24) | 2 (15) | 0.421 | 0.651 |
| 1.5 SD | 2 (25) | 1 (11) | 3 (20) | 1 (11) | 0.693 | 1.000 |
| 2.0 SD | 2 (29) | 2 (13) | 2 (13) | 1 (25) | 1.000 | 1.000 |
| **Visual disorder** |  |  |  |  |  |  |
| 1.0 SD | 1 (17) | 0 (0) | 9 (53) | 4 (31) | **0.048** | 0.064 |
| 1.5 SD | 1 (13) | 3 (33) | 7 (47) | 3 (33) | 0.306 | 0.321 |
| 2.0 SD | 1 (14) | 5 (33) | 6 (40) | 2 (50) | 0.734 | 0.346 |
| **Motor dysfunction** |  |  |  |  |  |  |
| 1.0 SD | 2 (33) | 0 (0) | 8 (47) | 6 (46) | 0.518 | 0.152 |
| 1.5 SD | 2 (25) | 2 (22) | 7 (47) | 5 (56) | 0.517 | 0.113 |
| 2.0 SD | 2 (29) | 6 (40) | 5 (33) | 3 (75) | 0.742 | 0.757 |
| **Communication deficit** |  |  |  |  |  |  |
| 1.0 SD | 2 (33) | 2 (40) | 6 (35) | 5 (39) | 1.000 | 1.000 |
| 1.5 SD | 2 (25) | 3 (33) | 5 (33) | 5 (56) | 1.000 | 0.519 |
| 2.0 SD | 3 (43) | 4 (27) | 6 (40) | 2 (50) | 0.749 | 0.533 |
| **Headaches** |  |  |  |  |  |  |
| 1.0 SD | 0 (0) | 1 (20) | 4 (24) | 4 (31) | 1.000 | 0.401 |
| 1.5 SD | 0 (0) | 3 (33) | 3 (20) | 3 (33) | 1.000 | 0.711 |
| 2.0 SD | 2 (29) | 4 (27) | 2 (13) | 1 (25) | 0.445 | 0.466 |
| **Seizures** |  |  |  |  |  |  |
| 1.0 SD | 0 (0) | 0 (0) | 2 (12) | 2 (15) | 1.000 | 0.559 |
| 1.5 SD | 0 (0) | 1 (11) | 1 (7) | 2 (22) | 1.000 | 0.629 |
| 2.0 SD | 1 (14) | 1 (7) | 2 (13) | 0 (0) | 0.615 | 1.000 |
| **Drowsiness** |  |  |  |  |  |  |
| 1.0 SD | 1 (17) | 1 (20) | 8 (47) | 6 (46) | 0.518 | 0.152 |
| 1.5 SD | 2 (25) | 2 (22) | 8 (54) | 4 (44) | 0.194 | 0.113 |
| 2.0 SD | 2 (29) | 4 (27) | 8 (53) | 2 (50) | 0.194 | 0.120 |
| **Itchy skin** |  |  |  |  |  |  |
| 1.0 SD | 3 (50) | 2 (40) | 11 (65) | 7 (54) | 0.524 | 0.489 |
| 1.5 SD | 4 (50) | 6 (67) | 8 (53) | 5 (56) | 1.000 | 1.000 |
| 2.0 SD | 3 (43) | 10 (67) | 8 (54) | 2 (50) | 1.000 | 0.758 |
| **Hair loss** |  |  |  |  |  |  |
| 1.0 SD | 1 (17) | 1 (20) | 6 (35) | 3 (23) | 0.476 | 0.694 |
| 1.5 SD | 1 (13) | 3 (33) | 5 (33) | 2 (22) | 0.491 | 0.736 |
| 2.0 SD | 0 (0) | 5 (33) | 5 (33) | 1 (25) | 0.491 | 0.725 |
| **Weakness of legs** |  |  |  |  |  |  |
| 1.0 SD | 3 (50) | 2 (40) | 9 (53) | 8 (62) | 1.000 | 0.725 |
| 1.5 SD | 3 (38) | 4 (44) | 8 (53) | 7 (78) | 1.000 | 0.216 |
| 2.0 SD | 3 (43) | 8 (53) | 9 (60) | 2 (50) | 0.746 | 0.756 |
| **Bladder control** |  |  |  |  |  |  |
| 1.0 SD | 0 (0) | 0 (0) | 7 (41) | 4 (31) | 0.151 | **0.020** |
| 1.5 SD | 1 (13) | 3 (33) | 5 (33) | 2 (22) | 0.491 | 0.736 |
| 2.0 SD | 1 (14) | 3 (20) | 6 (40) | 1 (25) | 0.272 | 0.290 |
| **Improvement** | **Improvement** | **Stable** | **Deterioration** | **Both** | **Improvement vs. others** | **Improvement/Both vs. others** |
| **QLQ-C30:** |  |  |  |  |  |  |
| **Global Health Status** |  |  |  |  |  |  |
| 1.0 SD | 3 (50) | 1 (20) | 5 (29) | 5 (39) | 0.393 | 0.346 |
| 1.5 SD | 3 (38) | 2 (22) | 4 (27) | 5 (56) | 1.000 | 0.189 |
| 2.0 SD | 3 (43) | 4 (27) | 5 (33) | 2 (50) | 0.673 | 0.463 |
| **Functional scales:** |  |  |  |  |  |  |
| **Physical** |  |  |  |  |  |  |
| 1.0 SD | 0 (0) | 2 (40) | 4 (24) | 3 (23) | 0.309 | 0.466 |
| 1.5 SD | 1 (13) | 4 (44) | 3 (20) | 1 (11) | 0.659 | 0.262 |
| 2.0 SD | 0 (0) | 5 (33) | 3 (20) | 1 (25) | 0.315 | 0.401 |
| **Role** |  |  |  |  |  |  |
| 1.0 SD | 4 (67) | 0 (0) | 6 (35) | 6 (46) | 0.187 | 0.120 |
| 1.5 SD | 5 (63) | 3 (33) | 5 (33) | 3 (33) | 0.225 | 0.518 |
| 2.0 SD | 4 (57) | 6 (40) | 4 (27) | 2 (50) | 0.401 | 0.287 |
| **Emotional** |  |  |  |  |  |  |
| 1.0 SD | 3 (50) | 2 (40) | 3 (18) | 5 (39) | 0.361 | 0.313 |
| 1.5 SD | 4 (50) | 2 (22) | 4 (27) | 3 (33) | 0.237 | 0.322 |
| 2.0 SD | 3 (43) | 4 (27) | 5 (33) | 1 (25) | 0.659 | 0.719 |
| **Cognitive** |  |  |  |  |  |  |
| 1.0 SD | 2 (33) | 1 (20) | 5 (29) | 6 (46) | 1.000 | 0.346 |
| 1.5 SD | 3 (38) | 2 (22) | 4 (27) | 5 (56) | 1.000 | 0.189 |
| 2.0 SD | 3 (43) | 4 (27) | 5 (33) | 2 (50) | 0.673 | 0.463 |
| **Social** |  |  |  |  |  |  |
| 1.0 SD | 5 (83) | 2 (40) | 5 (29) | 5 (39) | 0.066 | 0.216 |
| 1.5 SD | 6 (75) | 4 (44) | 4 (27) | 3 (33) | **0.049** | 0.335 |
| 2.0 SD | 5 (71) | 6 (40) | 6 (40) | 0 (0) | 0.105 | 1.000 |
| **Symptom scales** |  |  |  |  |  |  |
| **Fatigue** |  |  |  |  |  |  |
| 1.0 SD | 3 (50) | 2 (40) | 4 (24) | 3 (23) | 0.334 | 1.000 |
| 1.5 SD | 4 (50) | 4 (44) | 2 (13) | 2 (22) | 0.202 | 0.507 |
| 2.0 SD | 3 (43) | 6 (40) | 3 (20) | 0 (0) | 0.398 | 1.000 |
| **Nausea/vomiting** |  |  |  |  |  |  |
| 1.0 SD | 2 (33) | 1 (20) | 2 (12) | 2 (15) | 0.268 | 0.685 |
| 1.5 SD | 2 (25) | 1 (11) | 2 (13) | 2 (22) | 0.606 | 0.421 |
| 2.0 SD | 1 (14) | 3 (20) | 2 (13) | 1 (25) | 0.658 | 1.000 |
| **Pain** |  |  |  |  |  |  |
| 1.0 SD | 3 (50) | 1 (20) | 6 (35) | 3 (23) | 0.361 | 1.000 |
| 1.5 SD | 4 (50) | 2 (22) | 5 (33) | 2 (22) | 0.237 | 0.742 |
| 2.0 SD | 3 (43) | 3 (20) | 6 (40) | 1 (25) | 0.659 | 0.719 |
| **Dyspnea** |  |  |  |  |  |  |
| 1.0 SD | 1 (17) | 1 (20) | 3 (18) | 2 (15) | 1.000 | 1.000 |
| 1.5 SD | 2 (25) | 1 (11) | 3 (20) | 1 (11) | 0.606 | 1.000 |
| 2.0 SD | 1 (14) | 2 (13) | 3 (20) | 1 (25) | 1.000 | 1.000 |
| **Insomnia** |  |  |  |  |  |  |
| 1.0 SD | 3 (50) | 3 (60) | 10 (59) | 5 (39) | 1.000 | 0.354 |
| 1.5 SD | 5 (63) | 6 (67) | 7 (47) | 3 (33) | 0.697 | 0.756 |
| 2.0 SD | 3 (43) | 8 (53) | 8 (53) | 2 (50) | 0.697 | 0.734 |
| **Appetite loss** |  |  |  |  |  |  |
| 1.0 SD | 0 (0) | 1 (20) | 3 (18) | 2 (15) | 0.567 | 0.668 |
| 1.5 SD | 0 (0) | 1 (11) | 3 (20) | 2 (22) | 0.323 | 1.000 |
| 2.0 SD | 0 (0) | 1 (7) | 4 (27) | 1 (25) | 0.567 | 1.000 |
| **Constipation** |  |  |  |  |  |  |
| 1.0 SD | 2 (33) | 2 (40) | 5 (29) | 4 (31) | 1.000 | 1.000 |
| 1.5 SD | 3 (38) | 4 (44) | 4 (27) | 2 (22) | 0.692 | 1.000 |
| 2.0 SD | 3 (43) | 5 (33) | 5 (33) | 0 (0) | 0.659 | 1.000 |
| **Diarrhea** |  |  |  |  |  |  |
| 1.0 SD | 1 (17) | 2 (40) | 1 (6) | 0 (0) | 0.483 | 0.610 |
| 1.5 SD | 1 (13) | 2 (22) | 1 (7) | 0 (0) | 1.000 | 0.629 |
| 2.0 SD | 1 (14) | 3 (20) | 0 (0) | 0 (0) | 1.000 | 0.559 |
| **Financial difficulties** |  |  |  |  |  |  |
| 1.0 SD | 2 (33) | 3 (60) | 4 (24) | 2 (15) | 0.651 | 0.499 |
| 1.5 SD | 2 (25) | 4 (44) | 3 (20) | 2 (22) | 1.000 | 0.736 |
| 2.0 SD | 2 (29) | 4 (27) | 5 (33) | 0 (0) | 1.000 | 0.694 |
| **QLQ-BN20:** |  |  |  |  |  |  |
| **Future uncertainty** |  |  |  |  |  |  |
| 1.0 SD | 5 (83) | 2 (40) | 5 (29) | 5 (39) | 0.066 | 0.216 |
| 1.5 SD | 6 (75) | 3 (33) | 5 (33) | 3 (33) | **0.049** | 0.335 |
| 2.0 SD | 4 (57) | 6 (40) | 6 (40) | 1 (25) | 0.421 | 1.000 |
| **Visual disorder** |  |  |  |  |  |  |
| 1.0 SD | 2 (33) | 2 (40) | 4 (24) | 5 (39) | 1.000 | 0.737 |
| 1.5 SD | 4 (50) | 3 (33) | 3 (20) | 3 (33) | 0.237 | 0.322 |
| 2.0 SD | 3 (43) | 5 (33) | 4 (27) | 1 (25) | 0.389 | 0.719 |
| **Motor dysfunction** |  |  |  |  |  |  |
| 1.0 SD | 3 (50) | 2 (40) | 7 (41) | 3 (23) | 0.651 | 0.746 |
| 1.5 SD | 5 (63) | 1 (11) | 5 (33) | 4 (44) | 0.117 | 1.000 |
| 2.0 SD | 3 (43) | 6 (40) | 5 (33) | 1 (25) | 0.693 | 1.000 |
| **Communication deficit** |  |  |  |  |  |  |
| 1.0 SD | 2 (33) | 1 (20) | 4 (24) | 5 (39) | 1.000 | 0.493 |
| 1.5 SD | 4 (50) | 2 (22) | 4 (27) | 2 (22) | 0.202 | 0.507 |
| 2.0 SD | 3 (43) | 4 (27) | 4 (27) | 1 (25) | 0.398 | 0.701 |
| **Headaches** |  |  |  |  |  |  |
| 1.0 SD | 3 (50) | 1 (20) | 3 (18) | 3 (23) | 0.143 | 0.469 |
| 1.5 SD | 4 (50) | 2 (22) | 2 (13) | 2 (22) | 0.082 | 0.270 |
| 2.0 SD | 3 (29) | 4 (27) | 3 (20) | 1 (25) | 1.000 | 1.000 |
| **Seizures** |  |  |  |  |  |  |
| 1.0 SD | 1 (17) | 1 (20) | 1 (6) | 0 (0) | 0.386 | 1.000 |
| 1.5 SD | 1 (13) | 1 (11) | 1 (7) | 0 (0) | 0.488 | 1.000 |
| 2.0 SD | 0 (0) | 2 (13) | 1 (7) | 0 (0) | 1.000 | 0.551 |
| **Drowsiness** |  |  |  |  |  |  |
| 1.0 SD | 2 (33) | 1 (20) | 4 (24) | 4 (31) | 0.651 | 0.725 |
| 1.5 SD | 3 (38) | 4 (44) | 2 (13) | 2 (22) | 0.658 | 1.000 |
| 2.0 SD | 1 (14) | 6 (40) | 3 (20) | 1 (25) | 0.651 | 0.694 |
| **Itchy skin** |  |  |  |  |  |  |
| 1.0 SD | 2 (33) | 0 (0) | 1 (6) | 1 (8) | 0.095 | 0.321 |
| 1.5 SD | 2 (25) | 0 (0) | 2 (13) | 0 (0) | 0.165 | 1.000 |
| 2.0 SD | 1 (14) | 1 (7) | 2 (13) | 0 (0) | 0.542 | 1.000 |
| **Hair loss** |  |  |  |  |  |  |
| 1.0 SD | 1 (17) | 0 (0) | 4 (24) | 0 (0) | 0.567 | 0.350 |
| 1.5 SD | 1 (13) | 0 (0) | 4 (27) | 0 (0) | 1.000 | 0.382 |
| 2.0 SD | 0 (0) | 1 (7) | 4 (27) | 0 (0) | 0.567 | 0.300 |
| **Weakness of legs** |  |  |  |  |  |  |
| 1.0 SD | 1 (17) | 3 (60) | 2 (12) | 0 (0) | 1.000 | 0.191 |
| 1.5 SD | 1 (13) | 4 (44) | 1 (7) | 0 (0) | 1.000 | 0.373 |
| 2.0 SD | 1 (14) | 4 (27) | 1 (7) | 0 (0) | 1.000 | 1.000 |
| **Bladder control** |  |  |  |  |  |  |
| 1.0 SD | 2 (33) | 1 (8) | 3 (18) | 2 (40) | 0.578 | 0.703 |
| 1.5 SD | 3 (38) | 3 (33) | 2 (13) | 0 (0) | 0.172 | 1.000 |
| 2.0 SD | 1 (14) | 6 (40) | 1 (7) | 0 (0) | 1.000 | 0.412 |

Both: Both significant improvement and deterioration were observed in different NCF domains. NCF: neurocognitive function. QLQ-C30: European Organization for Research and Treatment of Cancer (EORTC) Quality of Life Questionnaire Core 30. QLQ-BN20: European Organization for Research and Treatment of Cancer (EORTC) Brain Cancer Module.
